# Therapeutic Administration of Recombinant Paracoccin Confers Protection against *Paracoccidioides brasiliensis* Infection: Involvement of TLRs

**DOI:** 10.1371/journal.pntd.0003317

**Published:** 2014-12-04

**Authors:** Ana Claudia Paiva Alegre-Maller, Flávia Costa Mendonça, Thiago Aparecido da Silva, Aline Ferreira Oliveira, Mateus Silveira Freitas, Ebert Seixas Hanna, Igor C. Almeida, Nicholas J. Gay, Maria Cristina Roque-Barreira

**Affiliations:** 1 Departamento de Biologia Celular e Molecular e Bioagentes Patogênicos, Faculdade de Medicina de Ribeirão Preto, Universidade de São Paulo, Ribeirão Preto, São Paulo, Brasil; 2 Border Biomedical Research Center (BBRC), Department of Biological Sciences, University of Texas at El Paso, El Paso, Texas, United States of America; 3 Department of Biochemistry, Cambridge University, Cambridge, United Kingdom; University of California, San Diego School of Medicine, United States of America

## Abstract

**Background:**

Paracoccin (PCN) is an *N*-acetylglucosamine-binding lectin from the human pathogenic fungus *Paracoccidioides brasiliensis*. Recombinant PCN (rPCN) induces a T helper (Th) 1 immune response when prophylactically administered to BALB/c mice, protecting them against subsequent challenge with *P. brasiliensis*. In this study, we investigated the therapeutic effect of rPCN in experimental paracoccidioidomycosis (PCM) and the mechanism accounting for its beneficial action.

**Methodology/Principal Findings:**

Four distinct regimens of rPCN administration were assayed to identify which was the most protective, relative to vehicle administration. In all rPCN-treated mice, pulmonary granulomas were less numerous and more compact. Moreover, fewer colony-forming units were recovered from the lungs of rPCN-treated mice. Although all therapeutic regimens of rPCN were protective, maximal efficacy was obtained with two subcutaneous injections of 0.5 µg rPCN at 3 and 10 days after infection. The rPCN treatment was also associated with higher pulmonary levels of IL-12, IFN-γ, TNF-α, nitric oxide (NO), and IL-10, without IL-4 augmentation. Encouraged by the pulmonary cytokine profile of treated mice and by the fact that *in vitro* rPCN-stimulated macrophages released high levels of IL-12, we investigated the interaction of rPCN with Toll-like receptors (TLRs). Using a reporter assay in transfected HEK293T cells, we verified that rPCN activated TLR2 and TLR4. The activation occurred independently of TLR2 heterodimerization with TLR1 or TLR6 and did not require the presence of the CD14 or CD36 co-receptors. The interaction between rPCN and TLR2 depended on carbohydrate recognition because it was affected by mutation of the receptor's *N*-glycosylation sites. The fourth TLR2 N-glycan was especially critical for the rPCN-TLR2 interaction.

**Conclusions/Significance:**

Based on our results, we propose that PCN acts as a TLR agonist. PCN binds to *N*-glycans on TLRs, triggers regulated Th1 immunity, and exerts a therapeutic effect against *P. brasiliensis* infection.

## Author Summary

*Paracoccidioides brasiliensis* is a pathogenic fungus that causes paracoccidioidomycosis (PCM) in humans, a debilitating fungal infection that mainly affects the lungs and is widespread in Latin America. Paracoccin (PCN) is a sugar-binding protein produced by this fungus. Previous studies have shown that PCN contributes to the colonization of host tissues by the fungus and induces the production of inflammatory factors (i.e., cytokines and nitric oxide) by immune cells such as macrophages. Here we investigated the therapeutic efficacy of recombinant PCN (rPCN) on the course of *P. brasiliensis* infection in mice. Histopathological analysis of lungs of animals treated with rPCN showed much lower inflammation in comparison to untreated, control mice. In addition, fewer infective *P. brasiliensis* yeast forms were recovered from the lung of rPCN-treated animals than from that of control animals. Administration of rPCN was associated with a profile of pro- and anti-inflammatory factors in the lung that was conducive to host protection. These effects were associated with PCN binding to sugar chains linked to innate immunity receptors, namely Toll-like receptors 2 and 4. These findings reveal a mechanism by which rPCN confers protection against PCM.

## Introduction

Paracoccidioidomycosis (PCM), first reported by Adolf Lutz in 1908 in Brazil, is an acute-chronic systemic mycosis caused by the dimorphic fungus *Paracoccidioides brasiliensis*. PCM is autochthonous to Latin America, and its incidence extends from southern Mexico to northern Argentina [1]. Infection is initiated by the inhalation of airborne propagules, derived from conidia, which transform into pathogenic yeast in the lung [2].

PCM infection can be asymptomatic or give rise to active disease. It causes pulmonary lesions, which lead to significant morbidity, with impairment of lung function. The disease can subsequently disseminate to other organs, producing secondary injuries to the mucosa, skin, lymphoid tissue, and adrenal glands. Acute and sub-acute PCM (juvenile form) develop within weeks to months and cause reticuloendothelial system hypertrophy; they are very severe and frequently mortal. The chronic type (adult form), which accounts for more than 90% of cases, primarily affects the lungs and progresses slowly, taking months to years to develop fully. PCM usually heals by fibrosis, which can permanently interfere with the patient's quality of life. In the absence of effective therapy, PCM can be lethal [3].

The immune response in infected individuals is primarily responsible for the clinical and pathological manifestations of PCM. In patients with active disease, cellular immunity [4], macrophage functions, and differentiation of Th1 cells [5] are often depressed. On the other hand, resistance to fungal infection is linked to the Th1-mediated immune response, which is triggered by the cytokine IL-12. Once secreted, IL-12 stimulates T lymphocytes to release high levels of interferon gamma (IFN-γ) [6]–[10].

The treatment of systemic mycoses frequently lasts one to two years. Currently, the antifungal drugs of choice are those derived from azole (ketoconazole and itraconazole), administered in association with sulfamethoxazole-trimethoprim, followed by sulfonamides and amphotericin B, for which patient relapse rates are high [11], [12]. Immunomodulatory agents able to stimulate balanced Th1 immunity can increase the efficacy of antifungals in experimental mycosis [13]. ArtinM, a d-mannose binding lectin, has immunomodulatory properties that protect against *P. brasiliensis* infection. Prophylactic and therapeutic protocols of ArtinM administration promote a Th1 immune response balanced by IL-10 [14]. A few studies have investigated the protective effect of antigens from the fungus itself, which are capable of inducing an effective cellular immune response and host protection [15]–[17]. Irradiated yeast cells confer long lasting protection in BALB/c mice, with a significant reduction in fungal burden in the lung, spleen, and liver [18]. Vaccination with a plasmid encoding the gp43-derived P10 peptide reduced fungal burden in the lung. Co-vaccination with a plasmid encoding mouse IL-12 yielded the best results in elimination of the fungus, with virtual sterilization at long-term infection. The immunization induced the production of IL-12 and IFN-γ [19].

Our group has shown that the recombinant form of paracoccin (rPCN) induces a Th1 protective response during PCM when prophylactically administered to mice [20]. Herein, we determined whether therapeutic administration of rPCN modified the course of experimental PCM. rPCN treatment drastically reduced pulmonary lesions and fungal burden and increased the pulmonary levels of Th1 cytokines and IL-10. Furthermore, stimulation of murine macrophages with rPCN induced IL-12 production, in a Toll-like receptor (TLR) 2- and 4-dependent manner, through recognition of TLR *N*-glycans by the rPCN carbohydrate-recognition domain (CRD).

## Materials and Methods

### Ethics statement

All *in vivo* experiments were approved by the Ethical Committee for Ethics in Animal Research (CETEA) of the School of Medicine at Ribeirão Preto, University of São Paulo. All efforts were made to minimize suffering, and the animal experiments were conducted in accordance with the Ethical Principles in Animal Research adopted by the Brazilian College of Animal Experimentation (COBEA) (Protocol 20/2013-1).

### Mice and the *P. brasiliensis* strain

Male BALB/c and C57BL/6 mice were used at 6–8 weeks of age. They were acquired from the vivarium on the campus of University of São Paulo at Ribeirão Preto, São Paulo, Brazil, and housed in the animal facility of the Molecular and Cellular Biology Department, Faculty of Medicine of Ribeirão Preto, University of São Paulo. Mice were maintained under optimized hygienic conditions.

The *P. brasiliensis* isolate, Pb18, was used to infect mice. The virulence of Pb18 yeast cells was maintained by periodic passages in mice, with subsequent recovery on brain-heart infusion (BHI) agar. Before experimental infection, yeast forms were grown in liquid BHI medium (HiMedia, Mumbai, India) for 3–7 days at 37°C with gentle agitation. Fungal cells were washed in sterile phosphate-buffered saline (PBS, pH 7.2) and counted in a Neubauer chamber. The viability of the yeast cells was determined by fluorescein diacetate and ethidium bromide staining [21]. Viability was always higher than 90%. Each experimental group consisted of five mice, and the assays were performed in triplicate.

### Purification of recombinant paracoccin (rPCN)

Recombinant paracoccin was expressed in *Escherichia coli*, as previously described [20]. It was purified by affinity chromatography on an *N*-acetylglucosamine column. Before use, endotoxins were removed with an immobilized polymyxin B agarose column (Bio-Rad, Hercules, CA, USA), according to the manufacturer's instructions. rPCN was then resuspended in endotoxin-free PBS (sterile PBS). For *in vitro* assays, rPCN aliquots were incubated for 1 h at room temperature (RT) with polymyxin (50 µg/mL; Sigma-Aldrich, St. Louis, MO, USA) to neutralize any potential contamination with bacterial lipopolysaccharides (LPS). rPCN preparations contained less than 0.05 ng/mL of bacterial endotoxin, as determined by the Endpoint Chromogenic LAL assay kit (Walkersville, Maryland, USA).

### Infection of mice and rPCN administration

BALB/c mice were infected with *P. brasiliensis* (1×10^6^ yeast in 100 µL PBS) by intravenous injection (i.v.) through the ophthalmic plexus. Uninfected control mice were inoculated with 100 µL PBS alone, under the same conditions as the infected group. After infection, mice were treated subcutaneously (s.c.) with rPCN (0.5 µg in 100 µL PBS). The treatment with rPCN was standardized according to number of administrations (1–3 doses). rPCN was administered as described in Table 1, after infection with *P. brasiliensis*. The course of infection was evaluated 30 days post-infection. In all experiments, the PBS used was sterile and endotoxin-free.

**Table 1:** Therapeutic groups.

Groups	rPCN administration (days post-infection)
Control^a^	3, 10, and 17
G1	3, 10, and 17
G2	3 and 10
G3	10
G4	3

a PBS alone.

### Histopathological analysis of the lung

Mice were euthanized 30 days post-infection. The lungs were excised, fixed in 10% formaldehyde for 24 h, dehydrated in ethanol, diaphanized in xylene, and embedded in paraffin. Histological sections were cut to a thickness of 5 µm and stained with hematoxylin-eosin. For morphometric and histological analysis, images were acquired at a magnification of 20× using a light microscope (Axiophot Photomicroscope; Carl Zeiss GmbH, Jena, Germany) with a camera (JVC TK-1270; Victor Company of Japan Ltd., Tokyo, Japan). Tissue and granuloma areas were determined using the ImageJ software (http://rsb.info.nih.gov/ij/), and the density was calculated as the ratio of the number of granulomas to tissue area (granulomas/mm^2^) in each section/mouse (6 mice per group). The total granuloma area (µm^2^) was determined by Σarea of sections/group. For visualization of the fungus in the granulomas, the tissue sections were stained with Gomori's methenamine silver.

### Fungal burden in the lungs of infected mice

Mice were euthanized 30 days post-infection and fungal burden was measured by colony-forming units (CFU). One lobe of the lung was aseptically removed from each animal, weighed, and homogenized in 1.0 mL sterile PBS using a tissue homogenizer (Ultra-Turrax T25 Basic; IKA Works, Inc., Wilmington, DE, USA). The final suspension (100 µL/Petri dish) was placed on solid BHI medium supplemented with 4% (v/v) heat-inactivated fetal calf serum (Invitrogen, Life Technologies, Camarillo, CA, USA). Gentamycin (Gibco, Grand Island, NY, USA) was added at 96 µg/mL. Petri dishes were incubated at 37°C for 7–14 days, and colonies were counted (1 colony = 1 CFU). The results indicated the number of viable *P. brasiliensis* colonies per gram of organ. They were expressed as the mean ± standard deviation (SD) and were representative of duplicate samples.

### Cytokine measurement

Lung homogenates were centrifuged at 2000× *g* for 15 min, at 4°C. The supernatants were collected and stored at −20°C. These samples were used to measure the levels of IL-12p40, IL-4, IL-10, tumor necrosis factor (TNF-α), and IFN-γ. The cytokines were detected by enzyme-linked immunosorbent assay (ELISA) using an OptEIA kit (Pharmingen, San Diego, CA, USA), according to manufacturer's instructions. Murine recombinant cytokines were used to create standard curves, and cytokine concentrations were determined with reference to the standard curves. The absorbance was read at 450 nm in a microplate scanning spectrophotometer (Power Wave-X; BioTek Instruments, Inc., Winooski, VT, USA). The results were expressed in pg/mL.

### Nitric oxide production

Nitric oxide (NO) production was quantified in the lung supernatants using the standard Griess reaction [22]. Briefly, fifty microliters of supernatant from the lung homogenates were distributed in 96-well microplates and incubated with 50 µL/well of Griess reagent (1.0% sulfanilamide, 0.1% naphthalenediamine dihydrochloride, and 2.5% H_3_PO_4_) at RT for 10 min. The absorbance at 550 nm was determined using a microplate reader. The absorbance was converted to micromolar (µM) NO with reference to a standard curve, generated using defined concentrations of NaNO_2_. All results were expressed in µM and are representative of triplicate experiments.

### Production of IL-12 by murine macrophages

C57BL/6 mice were i.p. injected with 2.0 mL sterile 3% sodium thioglycollate (Sigma-Aldrich). After 4 days, the animals were sacrificed, and their peritoneal cells were recovered by washing the cavity with 5.0 mL sterile PBS. The cell suspension was centrifuged at 300× *g* for 10 min and resuspended in Dulbecco's modified Eagle's medium (DMEM; Sigma-Aldrich) supplemented with 10% fetal bovine serum (FBS) (HyClone, Logan, UT, USA) and 1% penicillin-streptomycin (Gibco). Cells were plated in a 48-well culture plates (5×10^5^ cells/well) and incubated at 37°C in a 5% CO_2_ atmosphere overnight. Non-adherent cells were removed by washing in sterile PBS, and adherent cells were incubated in DMEM+10% FBS medium containing rPCN (2.5 µg/mL or 5.0 µg/mL). After 24-, 48-, and 72-h incubation periods, culture supernatants were harvested, and IL-12p40 levels were assessed by capture ELISA.

### Cell culture and transfection with TLR receptors, co-receptors, and TLR2 *N*-glycosylation mutants

Human embryonic kidney 293T (HEK293T) cells were cultured in DMEM (Sigma-Aldrich) supplemented with 10% FBS at 37°C in a humidified incubator with a 5% CO_2_ atmosphere. Cells were plated in 12-wells plates (5×10^5^ cells/well). After overnight incubation, cells (approximately 80% confluent) were transiently cotransfected with CD14, CD36, MD-2, and a combination of TLRs using Lipofectamine 2000 (Invitrogen, Carlsbad, CA, USA), as previously described [23]. The total amount of DNA per well was normalized to 2 µg by adding empty vector. All plasmids used for transfection were purified with the EndoFree plasmid kit (Qiagen, Chatsworth, CA, USA). After 24 h, the cells were transferred to 96-wells plates (4×10^4^ cells/well). Human TLR2 *N*-glycosylation mutant plasmids were generated as described by Weber et al. [24]. For transfection with these TLR2 mutants, HEK293T cells were plated in 96-wells plates (3.5×10^4^ cells/well). After 24 h, they were transiently transfected with constructs encoding TLR2 wild-type or mutant proteins. After overnight incubation, cells were stimulated for 20 h with the following positive controls: Pam3CysK4 (P3CSK4; EMC Microcollections, Tubingen, Germany) for TLR2/1, fibroblast stimulating ligand-1 (FSL-1; EMC Microcollections) for TLR2/6, and LPS-EB Ultrapure (standard LPS, *E. coli* 0111:B4; Sigma-Aldrich, St Louis, MO, USA) for TLR4, or with the negative control for cell stimulation (medium). Concentrations of 1.25 µg/mL or 5.0 µg/mL of rPCN were assayed. Cells transfected with empty vector, incubated with either medium or agonists (FSL-1 or P3C), were also assayed; negative results were required for including each system in this study. IL-8 was detected in the culture supernatants, using the OptEIA Human IL-8 ELISA kit (BD Biosciences). The results represent the mean ± SD, and the values are representative of triplicate experiments.

### Statistical analysis

Statistical analysis of the differences between the means of the experimental groups were performed by one-way analysis of variance (ANOVA), followed by Tukey's test using GraphPad Prism software (GraphPad Software, San Diego, CA, USA). All *in vivo* and *in vitro* experiments are representative of three independents assays, for which five animals were included per group. Differences with p<0.05 were considered statistically significant.

## Results

### Effect of therapeutic rPCN administration on pulmonary fungal load and histopathology

We first evaluated whether therapeutic administration of rPCN interfered with the course of PCM in a murine model, as previously reported for the prophylactic administration of rPCN [20]. Groups of BALB/c mice were i.v. infected with *P. brasiliensis* yeast and then submitted to different protocols of subcutaneous rPCN administration, which varied according to the number and timing of injections (see Table 1, Materials and Methods).

**Figure 1:**
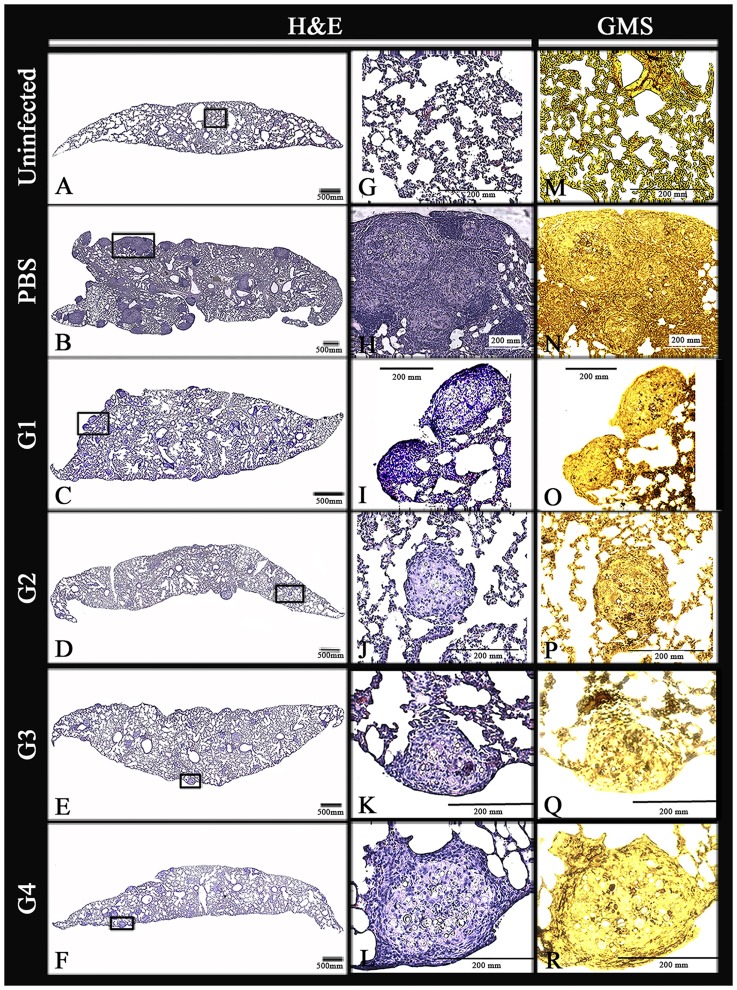
Lung histopathology of *P. brasiliensis*-infected mice treated with rPCN. The panels show representative lung sections from mice that were not infected (A); infected and not treated (B); or infected and therapeutically treated according to different protocols (C–F). The section were stained with hematoxylin and eosin (H&E) (panels G–L) or with Gomori's methenamine silver (GMS) (panels M–R). Images were captured using a Carl Zeiss Axiophot microscope coupled to a JVC TK1270 camera. Magnification bars = 500 µm for total lung and 200 µm for lung sections.

**Figure 2:**
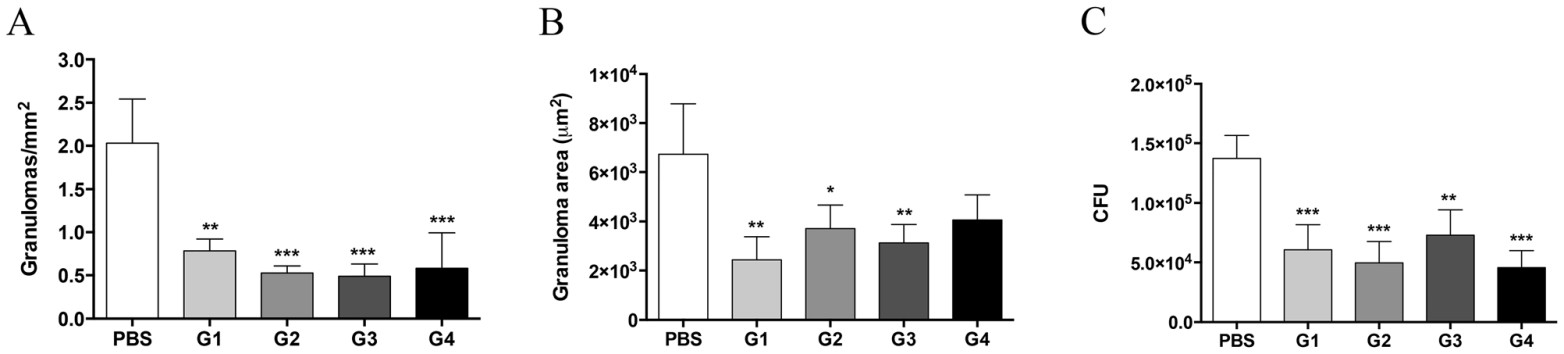
Morphometry of lung granulomas and analysis of fungal burden in infected animals treated with rPCN. Each group of mice was either not treated (PBS) or therapeutically treated according to protocols G1–G4 (see Material and Methods). Panel A: Number of granulomas per mm^2^ of tissue. Panel B: Granuloma area, in µm^2^. Panel C: Pulmonary CFU recovery. For morphometric analysis, the Image J program, developed by Wayne Rasband of the National Institute of Mental Health, was used. Bars depict the mean and SD. * p<0.05; ** p<0.01; *** p<0.001 *vs.* the PBS group.

Histopathological examination of the lung sections showed that organ architecture was preserved in rPCN-treated mice, with a few compact, individualized, and well-organized granulomas present, regardless of the administration regimen (Fig. 1C–F and 1I–L). On the other hand, severe lesions, characterized by a diffuse inflammatory reaction with several multifocal and coalescent granulomas, were observed in the lungs of untreated, control mice, which received only vehicle (PBS) (Fig. 1B and 1H). Morphometric analysis demonstrated that the number of granulomas in the lungs of rPCN-treated mice was lower than that in the lungs of the control mice (Fig. 2A). The granuloma density was 0.79, 0.53, 0.48, and 0.58 granulomas/mm^2^ of tissue for groups 1, 2, 3, and 4, respectively, compared with 2 granulomas/mm^2^ in the pulmonary tissue of control mice. This represents a difference of at least 60% between the number of granulomas present in the lungs of rPCN-treated mice and control mice. In addition, the total area occupied by granulomas in mice treated with rPCN on days 3, 10, and 17 post-infection (G1), on days 10 and 17 post-infection (G2), and day 10 post-infection (G3) was 2.8-, 1.8-, and 2.2-fold lower than the total area occupied by granulomas in control animals (Fig. 2B). Moreover, silver-stained sections showed that the number of yeast inside the granulomas was much larger in control mice than in rPCN-treated mice (Fig. 1N–R), suggesting that rPCN contributes to the control of fungal infection. The histopathology results were reinforced by the fungal burden analysis. CFU recovered from the lungs 30 days post-infection were significantly higher in the untreated control group than in the rPCN-treated groups: the pulmonary fungal burden was at least 50% lower in the treated groups (Fig. 2C). Taken together, our results demonstrate that therapeutic administration of rPCN is associated with the development of an appropriate inflammatory reaction and with the control of fungal burden, regardless of the inoculation regimen.

### Therapeutic rPCN administration augments the pulmonary levels of inflammatory mediators

**Figure 3:**
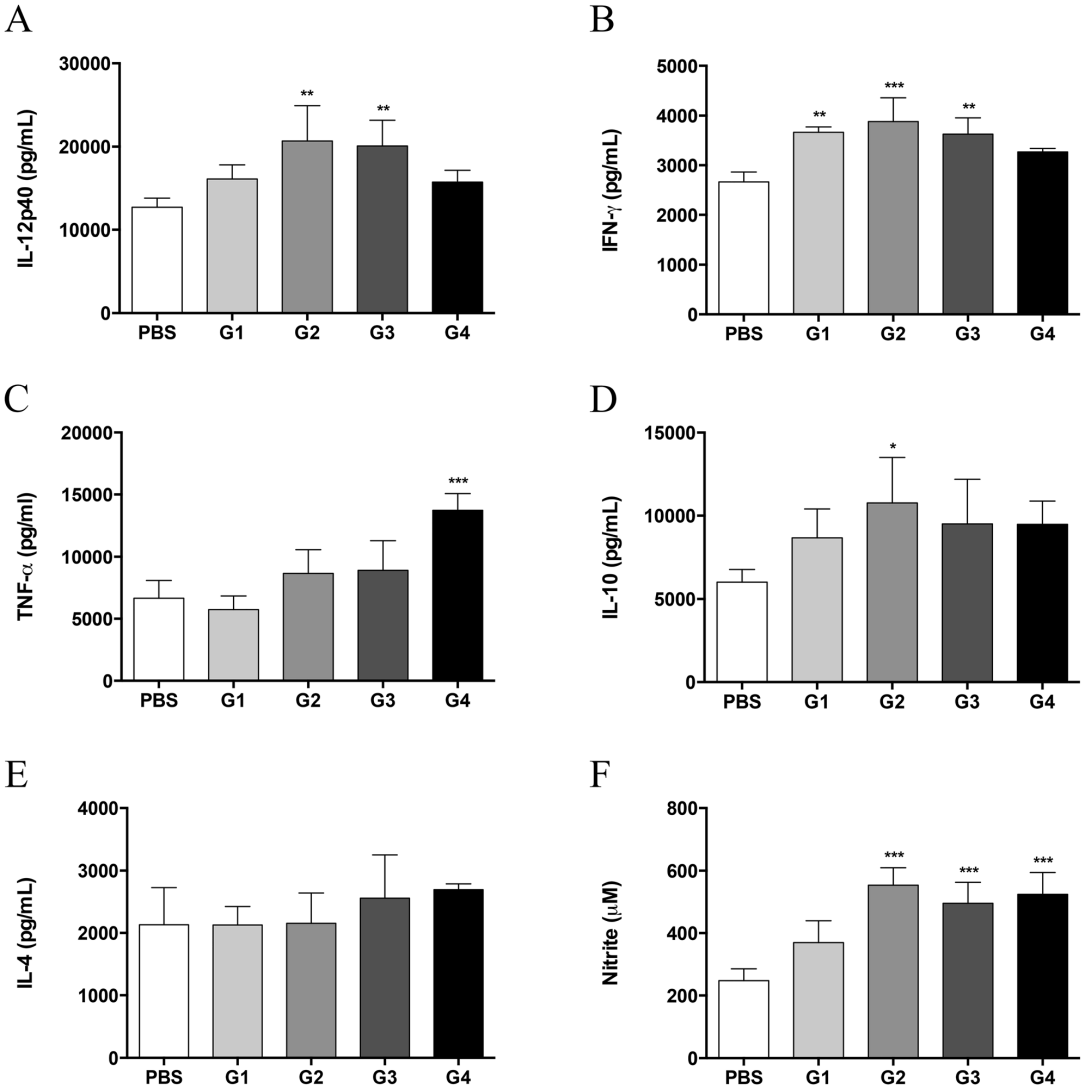
Therapeutic administration of rPCN increases proinflammatory cytokine and NO production. Lung homogenates were analyzed for IL-12p40 (A), IFN-γ (B), TNF-α (C), IL-10 (D), IL-4 (E), and NO (F) concentrations. Data represent the mean and SD of five mice per group; the experiments were performed in triplicate. * p<0.05; ** p<0.01; *** p<0.001 *vs.* the PBS group.

Because therapeutic rPCN administration protected against *P. brasiliensis* infection, we investigated the immune response profile of treated mice. Thirty days after infection, cytokine and NO levels in lung homogenates from treated mice and control mice were assessed. The highest levels of IL-12p40 (Fig. 3A) and IFN-γ (Fig. 3B) were detected in groups 2 and 3. Although modestly increased, IFN-γ levels in group 1 were significantly higher than those in control mice. The production of TNF-α (Fig. 3C), IL-10 (Fig. 3D), and IL-4 (Fig. 3E) was similar among the groups. Only groups 2 and 4 produced higher concentrations of IL-10 and TNF-α, respectively. In accordance with the observed inflammatory profile, NO levels in the lung homogenates of groups 2, 3, and 4 mice were higher than those of the untreated control mice (Fig. 3F). These results indicate that the protection against *P. brasiliensis* infection, conferred by therapeutic administration of rPCN, is associated with the development of Th1 immunity, as previously reported for prophylactic administration of rPCN [20].

### rPCN stimulates *in vitro* IL-12 production by murine peritoneal macrophages

**Figure 4:**
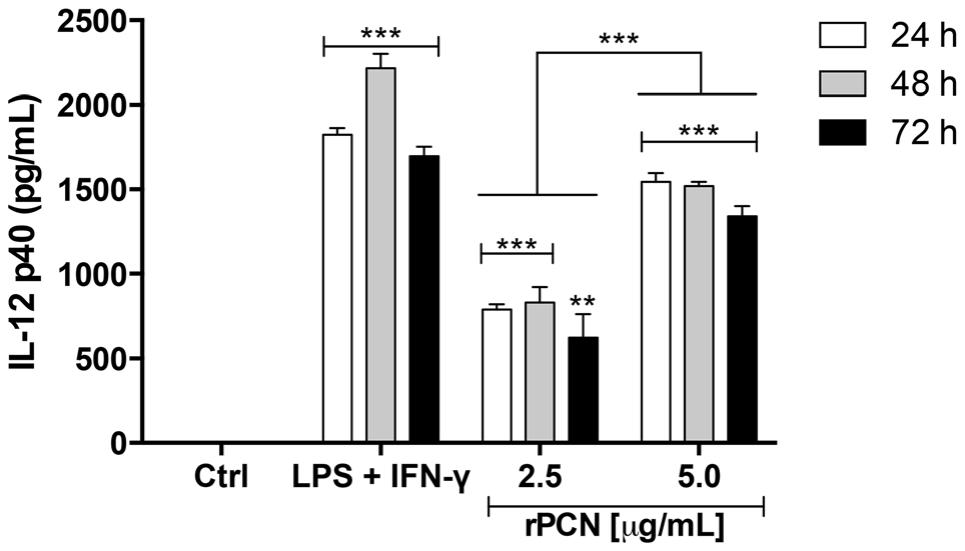
rPCN induces the *in vitro* production of IL-12p40 by murine macrophages. Cells were harvested from the peritoneal cavity of C57BL/6 mice stimulated with thioglycollate. Adherent cells were incubated with rPCN (2.5 µg/mL or 5.0 µg/mL) for 24, 48, and 72 h. Medium was used as a negative control; LPS+IFN-γ was used as a positive control. Tests were performed in triplicate. The statistical comparison was done between cells incubated with medium (negative control), cells stimulated with LPS+IFN-γ, and cells stimulated with rPCN (2.5 µg/mL and 5.0 µg/mL) for corresponding times. Statistical analyses were performed by one-way analysis of variance (ANOVA), followed by Tukey's multiple comparison test. The levels of IL-12 induced by rPCN were significantly lower when compared to the positive control, except after 72 h of incubation under the stimulus of 5.0 µg/mL rPCN; the levels of IL-12 induced by rPCN were significantly higher than that verified by the negative control; and, the IL-12 production was significantly higher when the cells were stimulated with 5.0 µg/mL rPCN in comparison with 2.5 µg/mL rPCN. The results were considered significant when p<0.01 (**) or p<0.001 (***).

Because rPCN administration led to the development of Th1 protective immunity in mice infected with *P. brasiliensis*, we examined IL-12p40 production by murine peritoneal macrophages stimulated with 2.5 or 5.0 µg/mL rPCN for 24, 48, and 72 h. Figure 4 shows that both concentrations of the lectin were able to induce the production of higher levels of IL-12 than the negative control. However, 5.0 µg/mL rPCN was more effective than 2.5 µg/mL rPCN in inducing this production. After 72 hours of incubation, the IL-12 levels induced by 5.0 µg/mL rPCN were similar to those induced by the positive control stimulus (LPS plus IFN-γ). On the other hand, the levels of IL-12 induced by 2.5 µg/mL rPCN were lower than that induced by the positive control, regardless of the incubation period.We hypothesized that the augmented production of IL-12 by macrophages could account for the Th1 protective response induced by rPCN *in vivo*, in which IFN-γ and TNF-α production conferred protection against PCM.

### rPCN triggers TLR activation

**Figure 5:**
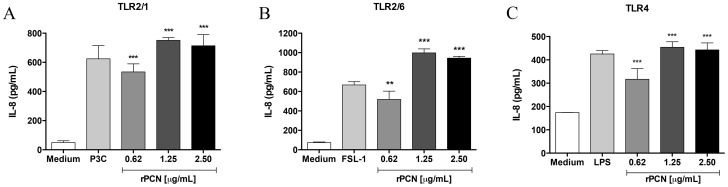
rPCN triggers TLR-mediated cell activation. HEK293T cells were transfected with CD14 and CD36 along with TLR2/1 (A), TLR2/6 (B), or TLR4 (C). The total amount of DNA in each transfection was kept constant by adding empty expression vector. The cells were stimulated with the indicated concentrations of rPCN, previously incubated with polymyxin to neutralize LPS, at 37°C for 20 h. The agonists used as positive controls were: Pam3CysSK4 (P3C) for TLR2/1, fibroblast stimulating ligand-1 (FSL-1) for TLR2/6, and bacterial lipopolysaccharide (LPS) for TLR4. Medium was used as negative control for cell stimulation (white bars). The cell supernatants were analyzed for IL-8 by ELISA. Statistical differences were determined by comparing transfected cells stimulated with rPCN to transfected cells incubated with medium. The results were considered significant when p<0.01 (**) or p<0.001 (***).

**Figure 6:**
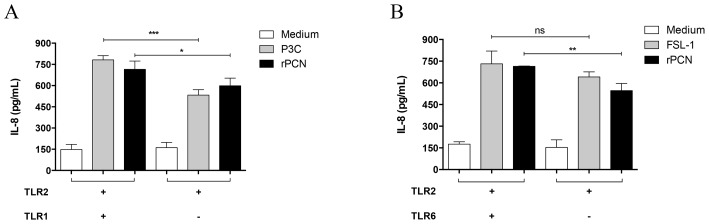
TLR2 heterodimerization is not critical for the cell activation triggered by rPCN. HEK293T cells were transfected with CD14 and CD36 along with TLR2, TLR2/TLR1 or TLR2/TLR6. The total amount of DNA in each transfection was kept constant by adding empty expression vector. After 48 h of transfection, cells were stimulated with the agonists: Pam3CysSK4 (P3C) for TLR2/1 and FSL-1 for TLR2/6. Medium was used as negative control for cell stimulation (white bars). rPCN (1.25 µg/mL), previously incubated with polymyxin to neutralize LPS, was assayed. The cell supernatants were analyzed for IL-8 by ELISA. Panel A: Cells transfected with TLR2 and TLR1. Panel B: Cells transfected with TLR2 and TLR6. Results are representative of five independent experiments. Statistical differences were assessed by comparing the response of cells expressing TLR2 to the response of cells expressing TLR2/TLR1 or TLR2/TLR6. Values are the mean ± S.D. * p<0.05; ** p<0.01; *** p<0.001.

**Figure 7:**
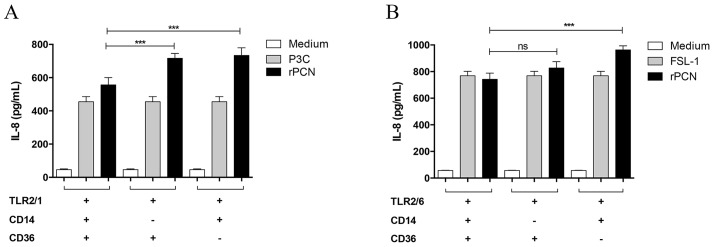
The absence of either CD14 or CD36 does not affect the TLR activation triggered by rPCN. HEK293T were cotransfected with CD14 and/or CD36 along with TLR2/1 (A) or TLR2/6 (B). The total amount of DNA in each transfection was kept constant by adding empty expression vector. The HEK293T cells were stimulated with rPCN (1.25 µg/mL), which was previously incubated with polymyxin to neutralize LPS. The positive controls were Pam3CysSK4 (P3C) for TLR2/1 and FSL-1 for TLR2/6. Medium was used as negative control for cell stimulation (white bars). Results are representative of three independent experiments. Statistical differences were assessed by comparing the response of cells lacking one of the co-receptors to the response of cells expressing both co-receptors, under similar stimuli. Values are the mean ± S.D. *** p<0.001.

IL-12 production by phagocytes is often initiated by the interaction of TLRs on the cell surface with pathogen-associated molecular patterns (PAMPs). Therefore, we next evaluated whether rPCN induced the activation of TLR4 and TLR2. HEK293T cells were transfected with plasmids encoding TLR2 heterodimers (TLR 2/1 or TLR2/6) or TLR4, and stimulated for 20 h with several concentrations of rPCN or with the appropriate TLR agonist (positive controls). Cell activation was assessed by measuring IL-8 levels in the culture supernatants by ELISA. rPCN triggered IL-8 production in TLR2/1 (Fig. 5A), TLR2/6 (Fig. 5B), and TLR4 (Fig. 5C) transfected cells. In all cases, rPCN at concentrations as low as 1.25 µg/mL was sufficient to trigger similar or higher responses than those induced by the control agonists. As shown in Figure 6, cells transfected with TLR2 alone decreased the IL-8 production in response to rPCN stimulus by 16% in the absence of TLR1 and 24% in the absence of TLR6. Thus, although rPCN interacted with homodimeric TLR2, heterodimerization of TLR2 with TLR1 or TLR6 modestly enhanced cell activation. Otherwise, in the absence of CD14 or CD36 co-transfection, the response of TLR2/TLR1- or TLR2/TLR6-transfected cells to rPCN was not reduced. This finding shows that TLR activation by rPCN is not affected by the absence of either CD14 or CD36 co-receptors (Fig. 7). These data suggest a mechanism by which rPCN protects animals during PCM.

### rPCN targets TLR2 through carbohydrate recognition

**Figure 8:**
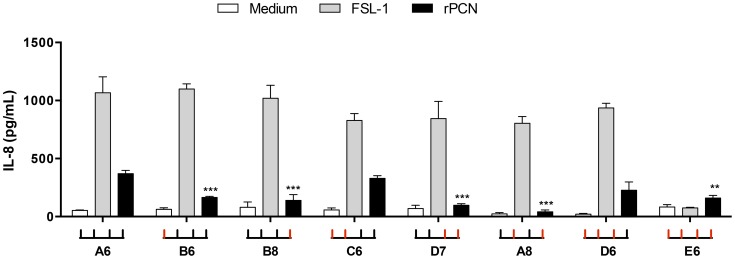
Reduction of r-PCN-induced activation of mammalian cells transfected with TLR2 glycosylation mutants. HEK293T cells expressing full-glycosylated TLR2 (WT – A6) or mutants of the N-glycosylation sites were stimulated with rPCN. The total amount of DNA in each transfection was kept constant by adding empty expression vector. Reduced activation was detected by comparing the IL-8 levels produced by HEK293Tcells expressing a certain TLR2 mutant with that produced by cells transfected with the full glycosylated TLR2. FSL-1 was used as a positive control. Medium supplemented with polymyxin (white bars) was used as negative control for cell stimulation. The E6 mutant was used as a negative control of the assay. For each transfected TLR2 ectodomain, the mutated site(s) [lacking N-glycans] is (are) represented by traces in red, while the preserved N-glycans are in black. Results are representative of two independent experiments. Values are mean ± S.D. The statistical comparison was done between HEK293T cells expressing full-glycosylated TLR2 (WT – A6) and the mutants of the N-glycosylation sites, stimulated with rPCN. The results were considered significant when p<0.01 (**); p<0.001 (***).

Because rPCN has a CRD able to bind GlcNAc, and TLR2 has four *N*-glycosylation sites in its ectodomain, we investigated whether the rPCN targeted the *N*-glycans on TLR2. HEK293T cells were transfected with plasmids encoding wild-type TLR2 or TLR2 mutants lacking one or more *N*-glycosylation sites [24]. Cells expressing wild-type or mutated TLR2 were incubated with rPCN for 20 h, and the levels of IL-8 in the culture supernatants were assessed. The level of IL-8 produced by cells transfected with the full-glycosylated TLR2, under rPCN stimulus, was compared with the levels produced by cells transfected with glycosylation mutants of TLR2 (B6, B8, C6, D7, A8, D6, and E6). The results are shown in Figure 8 as the IL-8 concentration in the supernatant of transfected cells. All these transfected cells, with exception of the E6 mutant, were responsive to the TLR2 agonist FSL-1, used as positive control. Medium, used as negative control, did not induce cell activation. The TLR2 mutants A8 (lacking the second and fourth N-glycans), D7 (lacking the third and fourth N-glycans), B6 (lacking the first N-glycans), B8 (lacking the fourth N-glycans), and E6 (lacking all four N-glycans) were associated with a significant decrease in the IL-8 production induced by rPCN, relative to the IL-8 production by cells expressing the wild-type TLR2 ectodomain (Fig. 8). The fourth *N*-glycan appears to be a critical target for rPCN CRD, once its presence, even in the absence of the three other *N*-glycans (D6 mutant), was sufficient to mediate rPCN-induced IL-8 production, at levels similar to those observed in cells expressing fully glycosylated TLR2.

Although E6 did not respond to the agonist (FSL-1), it worked as an appropriate negative control for the assay, once it was previously demonstrated that TLR2 lacking all four N-glycans is not secreted by the HEK cells (Weber, 2004).

## Discussion

Dramatic increases in the incidence of human fungal diseases worldwide as well as the toxicity and limited efficacy of anti-fungal drugs, especially without the help of host immune reactivity, require the development of new strategies for confronting fungal infections. Immunomodulation strategies are thought to hold promise. Their design entails a deep understanding of fungal interaction with various innate immune receptors [13], a necessity that became more obvious by the finding that an anti-fungal agent (amphotericin B) needs TLRs for efficacy [25]. Some exogenous lectins exert immunomodulatory effects in mammals by interacting with host-cell glycans [26]–[28], a fact that opens new perspectives in the design of strategies to control infectious diseases. ArtinM is the most studied immunomodulatory exogenous substance acting through carbohydrate recognition on cells of innate [23], [29]–[40] and adaptive [41] cells. Its effects favor host resistance against diseases caused by several pathogens [31], [36], [42], [43], including *P. brasiliensis* [14], [34], [44]. Nonetheless, as a plant lectin, ArtinM itself has restricted application in the prophylaxis or therapy of human diseases. On the other hand, paracoccin, as a lectin constituent of *P. brasiliensis*, the causal agent of PCM, could be potentially used for therapy against this endemic fungal disease in Latin America. This perspective has motivated us to characterize PCN effects in the course of PCM and elucidate the mechanisms that account for its property of inducing host Th1 immunity. This was discovered by administering PCN prophylactically to mice prior to be infected with *P. brasiliensis* yeasts. Remarkably, they became resistant to the infection, a fact that was attributed to the augmented pulmonary levels of pro-inflammatory mediators [20]. PCN was shown herein to be efficient also as a therapy against the ongoing mycosis, and the observed Th1 immunity was attributed to PCN interaction with N-glycans of TLR2 and TLR4.

The initial studies on PCN were performed by using a fraction obtained from *P. brasiliensis* yeast extracts, enriched by affinity chromatography onto d-GlcNAc-agarose columns. Since this d-GlcNAc-binding fraction stimulated the release of TNF-α and NO by murine macrophages [45], an immunomodulatory activity was conjectured for PCN. More recently, the availability of recombinant paracoccin has made possible the experimental validation of the original hypothesis. Our previous work that associated rPCN administration with Th1 immunity [20] is confirmed here, and is consistent with the fact that a mild or sub-clinical PCM infection, observed in resistant hosts, is linked to the release of Th1 cytokines by a subset of CD4^+^ T cells, such as IFN-γ and TNF-α [46]–[49]. Notably, rPCN administration on the days 3 and 10 after infection enhanced both Th1 immunity and IL-10 production. The mechanism by which IL-10 might work in this model of infection is discussed below.

The development of Th1 lymphocytes depends on stimulation by IL-12 [49], a cytokine that is mainly derived from activated cells of the innate immune system [50]. In the present study, we verified that rPCN stimulates murine macrophages to augment IL-12 production, a finding that led us to hypothesize that like Artin M [24], rPCN can interact with TLRs on the macrophage surface. This hypothesis was confirmed by signaling assays in HEK293T cells transfected with TLR2 or TLR4, in which rPCN caused TLR-mediated cell activation, as manifested by augmentation of IL-8 production [51], [52]. Most TLR2 agonists are recognized by receptor heterodimers, formed by association of TLR2 with TLR1 or TLR6 [50]–[52]. In addition, the participation of co-receptors is often required for efficient activation. Using the same cell-based assay, we show that rPCN could trigger TLR2 activation independently of heterodimerization, and that the cell response does not require CD14 or CD36 to be stimulated. The hypothesis that rPCN interacted with N-linked glycans of the TLR ectodomain was validated by using, specifically, HEK293T cells transfected with TLR2 mutants, in which the N-glycosylation sites were successively disrupted [24]. This system has revealed that the TLR2-rPCN interaction was dependent on carbohydrate recognition and that the fourth TLR2 N-glycan was critical for establishing the interaction and triggering cell activation. It was previously reported that the N-glycosylation status is essential for secretion and function of TLRs, including TLR2 [24] and TLR4 [53], a fact that is consistent with the high degree of conservation of TLR N-glycosylation sites between species [24]. Concerning TLR2, Weber et al. (2004) [24] reported that monoglycosylated and diglycosylated mutants do not support secretion. Intriguingly, our data indicate that rPCN, as well as positive control agonist FSL1, triggers activation of HEK293T cells expressing some mono- (D6) and diglycosylated (A8, D7, and C6) TLR2 mutants. Our group obtained similar results when studying the recognition of TLR2 *N*-glycans by the lectins r*Tg*MIC1 e r*Tg*MIC4 of *Toxoplasma gondii*, which also activated HEK293T cells expressing mono- and diglycosylated mutants (unpublished data).

The production of Th1 cytokines and the concomitant increase in the pulmonary levels of IL-10 following the rPCN administration can be explained by TLR pathway activation. Inflammatory signaling by TLRs results in the downstream activation of NF-κB, IFN regulatory factors (IRFs), and MAPKs. In addition, ITAM signaling and IFN-γ, whose production is highly augmented in mice treated with rPCN, cooperate with the crosstalking among the macrophage activation pathways favoring a balance between pro- and anti-inflammatory cytokines, with IL-10 and Stat3 involved in TLR-induced feedback inhibition. The mechanism involves GSK3, AP-1, CREB, and Akt as major regulators of the TLR2-induced feedback-inhibition loop, with IFN-γ suppressing this mechanism [54]. Ultimately, the balance in cytokine production would prevent severe immunopathology that could occur in response to rPCN administration, a distinctive feature of the protective effect of rPCN against *P. brasiliensis* infection.

**Figure 9:**
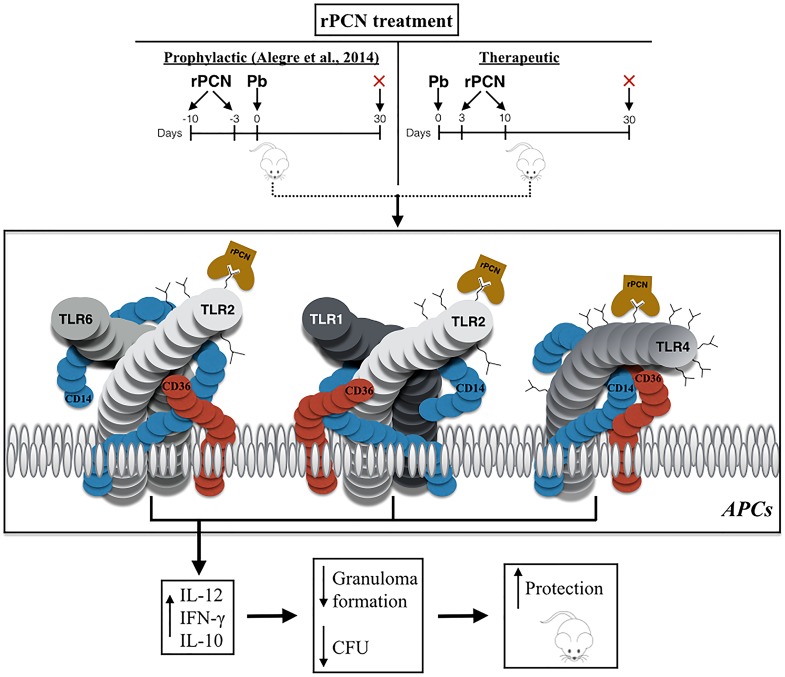
Possible mechanism of the protection against murine paracoccidioidomycosis conferred by paracoccin administration, as suggested by *in vivo* and *in vitro* studies. Once administered to BALB/c mice, before or after inoculation of *P. brasiliensis* yeasts, rPCN interacts with N-glycans of TLRs on antigen-presenting cells (APC). It triggers IL-12 production, which drives immunity to the Th1 axis. Production of IL-10 is also induced. The consequent balanced Th1 immunity that is developed protects mice against PCM, as manifested by lower incidence of granulomatous lesions and more efficient fungal clearance in the lungs, at day 30 post-infection.

Our results and assumptions allow us to delineate a sequence of events that can constitute the mechanism of rPCN effects *in vivo* (Figure 9). Once administered to mice, rPCN interacts with TLRs, induces IL-12 production, thereby driving immunity to the Th1 axis, with a balanced bias due to concomitant production of IL-10, and modification of the course of experimental PCM.

In conclusion, our study demonstrates that therapy with recombinant paracoccin provides resistance against *P. brasiliensis* infection by inducing balanced Th1 immunity, which is triggered by the lectin interaction with TLR2 N-glycans. Moreover, it addresses the challenge of identifying fungal antigens that can induce optimal immune responses *in vivo* by targeting innate immunity receptors on antigen presenting cells. Therefore, paracoccin may have its use as an appropriate immunotherapy for paracoccidioidomycosis.
